# Sustained multi-year contraceptive efficacy of single-dose GonaCon in synanthropic female capybaras

**DOI:** 10.1590/1984-3143-AR2025-0142

**Published:** 2026-04-20

**Authors:** Derek Andrew Rosenfield, Cristiane Schilbach Pizzutto

**Affiliations:** 1 Departamento Reprodução Animal (Animais Silvestres), Faculdade de Medicina Veterinária e Science Animal, Universidade de São Paulo, São Paulo, SP, Brasil; 2 Laboratório de Ecologia e Evolução, Instituto Butantan, São Paulo, SP, Brasil

**Keywords:** capybara, immunocontraception, GonaCon, anti-GnRH, fertility control, Brazilian Spotted Fever

## Abstract

Capybaras (*Hydrochoerus hydrochaeris*) are increasingly synanthropic throughout South America, where their rapid population growth in human-altered landscapes heightens conflicts and, critically, elevates the public health risk of Brazilian Spotted Fever (BSF), a lethal *Rickettsia rickettsii* zoonosis transmitted by *Amblyomma sculptum* ticks. Since capybara density is tightly coupled with pathogen circulation, non-lethal, scalable management strategies are essential. We evaluated the long-term efficacy and safety of the single-dose GonaCon™ immunocontraceptive vaccine in free-living, sexually mature female capybaras (n=6 treated, n=3 control) ~ 33 months. All treated females demonstrated sustained reproductive suppression (absence of parturition or estrus behavior; *p* < 0.01). Efficacy was confirmed by multi-modal evidence: plasma progesterone (P4) and estradiol (E2) concentrations were significantly suppressed, showing reductions of 41% and 36% by Day 27 post-vaccination, respectively (*p* < 0.005; Cohen’s *d* > 1.5). Histological analysis validated HPG axis disruption, revealing significantly lower ovarian weights (*p*=0.002) and suppressed folliculogenesis, with treated ovaries possessing drastically fewer antral follicles (mean 2.3 ± 0.5 vs. 12.7 ± 1.2 in controls; *p*<0.001). The treatment was safe, with the only adverse effect being a transient, localized swelling in 4/6 animals that resolved within two weeks. Importantly, treated females maintained critical social behaviors, including alloparental care. These findings provide strong evidence for the sustained, multi-year efficacy of GonaCon in a large wild rodent, supporting its use as a humane, non-surgical tool for density management and BSF risk mitigation under the One Conservation Paradigm.

## Introduction

The successful adaptation of the world’s largest rodent, the capybara (*Hydrochoerus hydrochaeris*), to human-modified habitats, high fecundity, abundant food resources, and the virtual absence of natural predators has led to localized population overabundance throughout South America (Abreu Bovo et al., 2016; Verdade and Ferraz, 2006). The resulting human-wildlife conflicts (Ferraz et al., 2003) are overshadowed by the species’ critical role as an amplifying host for the tick vector (*Amblyomma sculptum*) of Brazilian Spotted Fever (BSF) (Labruna, 2013; Krawczak et al., 2014; Brites-Neto et al., 2015). Brazilian federal laws restrict the use of lethal control for native, protected wildlife species (Rosenfield et al., 2019a), necessitating the adoption of humane, non-surgical, and scalable management strategies (Alvarez and Kravetz, 2006).

Currently, two non-lethal fertility-control strategies are practically available for capybaras. Surgical sterilization includes techniques such as uterine horn ligation (Passos-Nunes et al., 2024) and Ovarian Sparing Spay (OSS) variants (Yanai et al., 2022), offering permanent efficacy. The same advances in minimally invasive surgical sterilization techniques further demonstrate the feasibility of permanent fertility control in other free-ranging wildlife, including mini-laparotomy successfully applied in South American coatis (*Nasua nasua*) (Penido et al., 2024). Immunocontraception, represented by the GonaCon™ vaccine (Miller et al., 2008), is valued for its single-dose formulation and multi-year efficacy (National Research Council, 2013), proven in cervids (Gionfriddo et al., 2008; Killian et al., 2009; Powers et al., 2014), equids, marsupials, and suids (Massei et al., 2012; Wimpenny et al., 2021). The duration of efficacy in rodents is highly variable (Jacob et al., 2008; Krause et al., 2014; Pai et al., 2011), ranging from 1–2-year reversal in some prairie dogs (Yoder and Miller, 2010) to prolonged suppression in others (Shiels et al., 2024). A comprehensive overview of GonaCon applications in rodents, including efficacy, duration, and safety profiles, is provided in Supplementary Table D.

Prior work confirmed GonaCon’s efficacy in male capybaras (Rosenfield et al., 2019a), but the core demographic driver, the female, remained unstudied. Furthermore, any intervention must preserve the capybara's gregarious behavior, especially alloparental care (Rosenfield et al., 2019b), a key social role.

This study evaluates the long-term (≥ 33-month) efficacy, endocrinological, social impact, and safety of a single GonaCon dose in free-living female capybaras. This research is framed within the One Conservation Paradigm (Pizzutto et al., 2021).

## Methods

The study was conducted from January 2016 to August 2019 at the University of São Paulo main campus (23°33′21″S, 46°43′14″W; altitude 722 m). The 247.5 ha study area comprised open grassland surrounding a man-made lake originally built for aquatic sports. This riparian habitat supported a synanthropic capybara population. The local vegetation provided critical resources, including riparian grasses (*capim*), common Atlantic Forest/Cerrado fauna, and cultivated/introduced plants such as banana (*Musa* spp.) and sugarcane. The climate is humid subtropical with two distinct seasons: wet (October–March; mean temperature 25–30°C; median rainfall ~170 mm/month) and dry (April–September; mean temperature ~19°C; rainfall ≤50 mm/month) (WMO, 2019).

### Study population history

The local capybara population originated from a single founding pair in 2013 and expanded to over 40 individuals by 2016, forming two stable social groups and supporting several solitary males (satellites) (Table 1). A total of nine sexually mature females (estimated mean body weight 60 kg) were targeted for treatment.

**Table 1:** Population Status Quo Pre-Project 1^st^ semester 2016.

**Group**	**Adult (alpha) Male**	**Adult Females (dominant / subordinate)** Sexual maturity: ~ 18 months (females) ~ 24 months males	**Juveniles/sub adults** Sexually immature ~4 months ~12 months Subadult: 12–18 months	**Pups (Neonate/Infant)** Birth ~4 months old
**Group I**	1	14	15	5
**Group II**	1	5	1	
**Satellite Solitary Males**	3			

### Capture, handling, and study design

**Figure 1:**
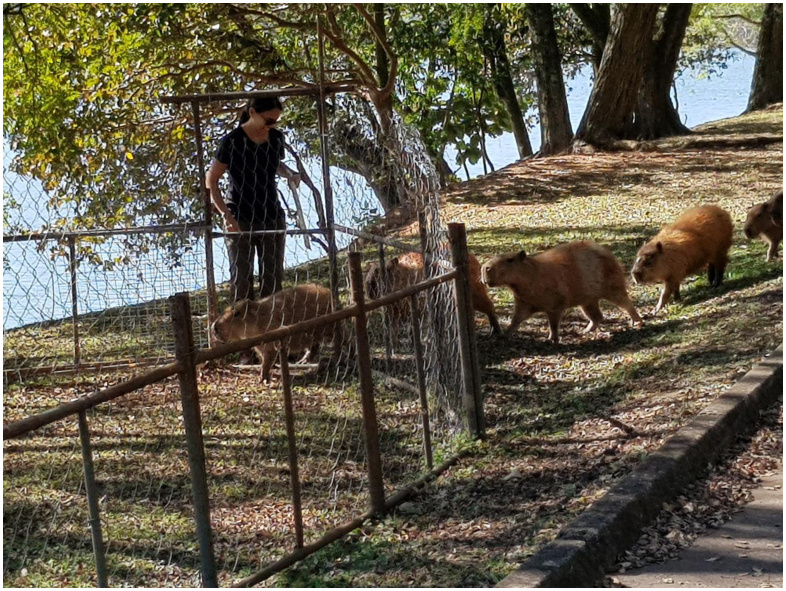
Controlled confinement via positive reinforcement to mitigate capture stress.

To ensure minimal stress and maximum safety during the intervention, the capybara groups were not immediately chemically immobilized in the field. Instead, the study utilized positive-reinforcement operant conditioning (Rosenfield and Pizzutto, 2019c). This conditioning allowed the research team to safely gather the targeted groups into pre-positioned enclosed confinement (Figure 1) areas prior to any physical or chemical manipulation.

This critical step served a dual purpose: it reduced the stress associated with the intervention, chemical restraint and most importantly prevented the risk of recently sedated animals escaping into adjacent water bodies, thereby eliminating the significant welfare risk of drowning during the induction or recovery phase of anesthesia. Once safely confined, chemical immobilization was administered for the subsequent health evaluation, vaccine injection and biological data collection.

Photograph illustrating the use of an enclosed confinement area during the handling protocol. Animals were operant conditioned using sugarcane (bait) to voluntarily enter this area, ensuring a calm pre-sedation environment. This method was critical for minimizing procedural stress and eliminating the risk of drowning during chemical induction, which is a known welfare concern in this species. Source: © 2019 D. Rosenfield.

Furthermore, this control strategy mitigated critical welfare risks inherent to field capture. This confinement allowed for the necessary expeditious treatment in case of acute complications. As hindgut fermenters, capybaras consuming bait high in fermentable carbohydrates (e.g., sugarcane) combined with the use of anesthetic protocols are at risk of a pharmacological depression of visceral motility. This inhibition can rapidly lead to the accumulation of fermentation gases and the onset of the life-threatening condition, Acute Cecal Tympany (Rosenfield et al., 2019d). The controlled confinement transformed the procedure from a high-risk event into a controlled veterinary manipulation, ensuring animal stabilization and the prompt management of this known iatrogenic risk.

Between May and August 2017, nine sexually mature females (estimated mean body weight 60 kg) were remotely immobilized using CO_2_-powered tranquilizer darts (Pneu-Dart, USA) containing ketamine (9 mg/kg; Syntec, Brazil) and dexmedetomidine (5 µg/kg; Zoetis, Brazil) (Rosenfield et al., 2020). Individuals were clinically examined, weighed, microchipped, and marked with colored ear tags, identifying GonaCon or Sham vaccine treated. After evaluation, all nine sexually mature female capybaras (*Hydrochoerus hydrochaeris*), all confirmed as multiparous, were selected for the study.

### Group allocation

Despite observing capybara population with over 40 individuals, the study utilized a selection process based on a rigorous filtering criterion derived from the capybara's strict social structure. Given that reproduction in the main group is largely confined to the highest-ranking, multiparous females, those retaining reproductive rights and actively contributing to current population growth, only individuals confirmed as sexually mature, dominant, and visibly multiparous were selected for the study. This methodology ensured that every female included in the fertility control trial represented a primary, known reproductive driver within the local population dynamics, maximizing the power of the efficacy data gathered despite the low overall numerical sample. From the nine captured individuals, nine multiparous females were selected as study subjects. From all the females that fulfilled the criteria, the assignment (Table 2) was performed at random to maximize scientific rigor and minimize selection bias.

**Table 2:** Group allocation.

**Group**	**Treatment**	**n**	**Injection Site**
**Treated Group**	**GonaCon™** (USDA/APHIS NWRC)	**6**	Intramuscularly into the semimembranosus/semitendinosus region.
**Control Group**	**Sham Vaccine** (Placebo with identical adjuvant)	**3**	Same anatomical site.

### Justification of sample size and randomization

Due to logistical, financial, regulatory, and ethical constraints inherent in wildlife research, a small total sample size (n=9) and a 6:3 group split was employed (Supplementary Table A).

### Endocrine analysis

Due to the constraints of field capture, basal blood samples were collected pre, - and post vaccination in staggered dates. Samples were centrifuged, and serum was stored at -80°C until analysis. The samples were quantified via validated commercial ELISA kits for progesterone (P4) and estradiol (E2).

### Histopathological and morphometric analysis

At the conclusion of the ~ 33-month monitoring period, all animals were recaptured for ovariectomy. Ovaries were weighed, measured, and fixed in 10% formalin for H&E staining to assess ovarian and parenchymal morphology, as well as folliculogenesis by counting primary, secondary, and antral follicles.

### Behavioral monitoring

Focal behavioral monitoring was conducted biweekly from August 2016 to August 2019 (six months pre-treatment and 33 months post-treatment), using a prior developed ethogram, identifying social interaction, alloparental care (e.g., nursing, pup herding), and more specific reproductive indicators:

- estrous receptivity and copulatory behavior
- isolation prior to parturition
- teat morphology (non-lactating ~10 mm vs lactating/visibly swollen ~25 mm).

These indicators were quantified during all monitoring events. Observations (10–50 m distance) were performed with binoculars and high-definition cameras.

### Ultrasound-assisted pregnancy monitoring

Opportunistic ultrasound examinations were performed on females during handling events to assess potential pregnancies, providing supplemental and non-systematic pregnancy monitoring throughout the study.

### Statistical analysis

Comparisons between groups were performed using Welch’s t-test, appropriate for unequal variances and small sample sizes (Delacre et al., 2017), (n=6 treated, n=3 controls). Proportional fertility data were analyzed using Fisher’s Exact Test. Cohen's *d* was calculated to report effect sizes. The alpha level was set to *p* < 0.05.

### Approvals

All procedures were approved by the Brazilian Ministry of Environment (SISBio, 2016) and the University of São Paulo Ethics Committee (CEUAVET, Protocol 95532608162016). Genetic heritage registration was obtained via SisGen AD1B160.

GonaCon, in Brazil, is not commercially available. The import of the non-registered GonaCon vaccine for this research was specifically authorized by the Brazilian Ministry of Agriculture, Livestock, and Food Supply (MAPA) under *Licenciamento de Importação* (LI: 16/3557599-5) for scientific experimentation.

## Results

### Sustained efficacy and reproductive monitoring

The single GonaCon dose provided sustained and complete reproductive suppression in all six treated females over the entire 33-month monitoring period, with zero parturitions recorded. The control group had confirmed litters during this period (*p* < 0.01).

### Endocrinological assessment and confirmation of HPG axis suppression

**Figure 2:**
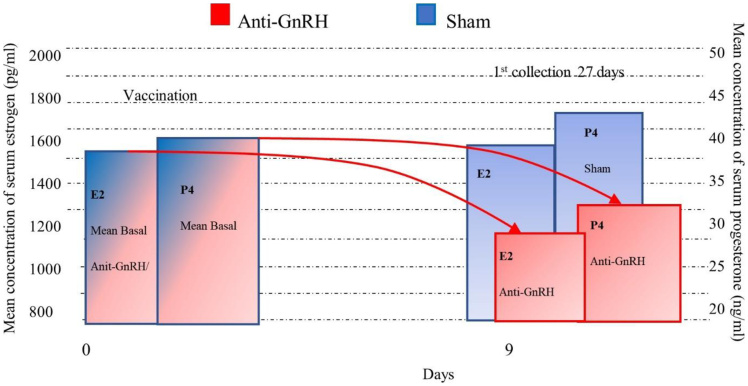
Time-Course Suppression of Plasma Progesterone (P4) Following Single-Dose GonaCon Immunization. Mean (± SEM) plasma concentrations of Progesterone (P4 ng/mL) and Estradiol (E2 pg/mL) measured over 27 days. GonaCon-treated females (n=6), compared to Sham-treated control females (n=3) presenting sustained suppression of both P4 and E2 plasma levels post-treatment, confirming the functional immunological disruption of the entire Hypothalamic–Pituitary–Gonadal (HPG) axis.

GonaCon resulted in rapid and significant suppression of reproductive hormones Figure 2:

- **Progesterone (P4):** Dropped by **41.1%** at Day 27 (*p*=0.005).
- **Estradiol (E2):** Decreased by **36.0%** at Day 27 (*p*=0.008).

### Body weight

Pre-treatment body weights were similar between groups (Table 3; p=0.629). No treated females displayed estrous behavior or produced offspring over 33 months, vs. 2/3 controls with confirmed litters (p<0.01, Fisher’s exact).

**Table 3:** Pre-vaccination body weight (Treatment vs. Sham Groups).

**Group**	**n**	**Body Weight (kg) ± SEM (SD)**	**p-value**	**Reference (Wild Capybaras)**
**Control**	3	69.6 ± 5.1 (8.8)	0.629	~55 kg (Ojasti, 1973; Miglino et al., 2013)
**Treated**	6	66.6 ± 2.4 (5.9)		

Pre-treatment body weights (mean ± SEM/SD). Synanthropic capybaras exhibit greater mass than wild counterparts.

### Ovaries

Ovarian morphometry showed significant weight reduction in treated females (Table 4; p=0.002), with no differences in length/width (p>0.05).

**Table 4:** Ovarian Morphometry (Control vs. Treated; Mean ± SEM/SD)

**Parameter**	**Control (n=3)**	**Treated (n=6)**	**p-value**	**Reference (Wild)**
Length (mm)	30.37 ± 0.08 (0.14)	30.91 ± 0.52 (1.27)	0.219	~19 mm (Ojasti, 1973)
Width (mm)	16.92 ± 1.77 (3.07)	16.24 ± 1.22 (3.00)	0.683	~15 mm (Miglino et al., 2013)
Weight (g)	3.55 ± 0.20 (0.35)	2.29 ± 0.08 (0.20)	0.002	2.9 g (Miglino et al., 2013)

Ovarian Histopathology.

**Figure 3:**
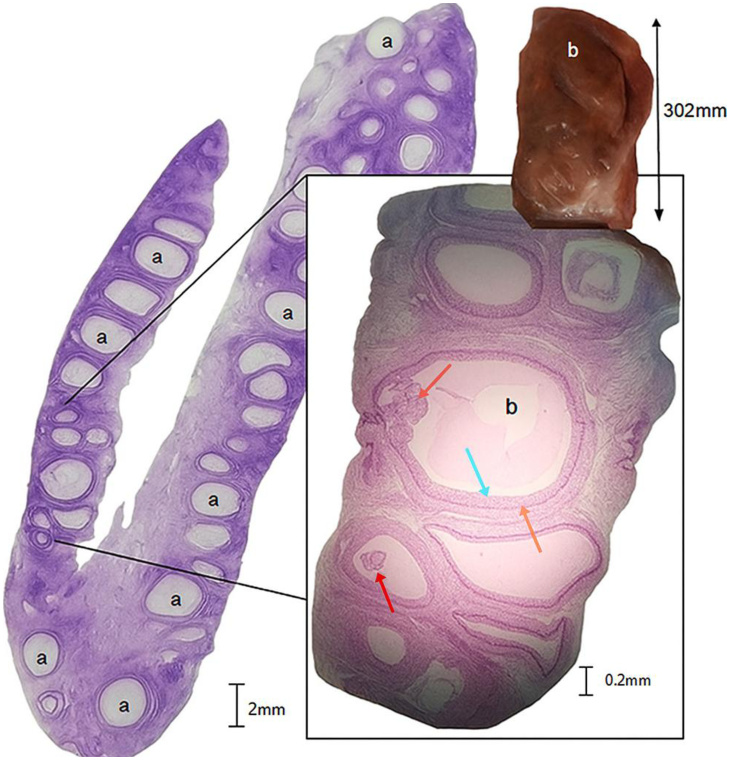
Histological section from control ovary. Tertiary follicle with intact granulosa cells and visible oocyte (H&E, 400×). Longitudinal cut, hematoxylin and eosin staining. Depicting (a) large number of antral follicles; (b) magnified section, showing a tertiary follicle with well-defined follicular antrum; Red arrow indicating developing antral follicle with oocyte and visible corona radiata. Blue arrow: granulosa cells; green arrow: theca cells, and orange arrow: remanence of granulose cells, forming the cumulus oophorous and corona radiata, w/o oocytes in this section; (c) elongated oval-shaped ovary with protrusions and an uneven surface. Source: © 2019 D. Rosenfield

**Figure 4:**
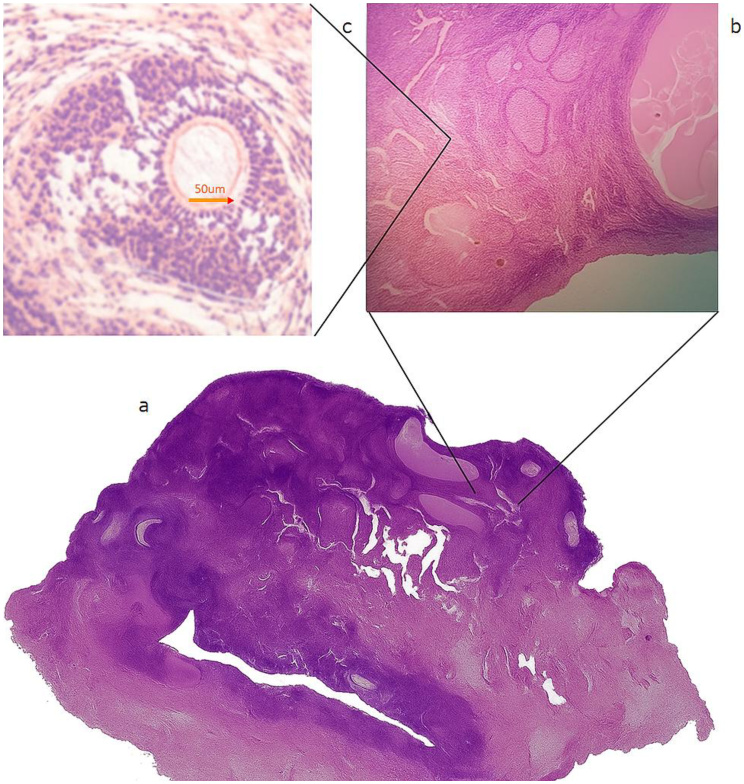
Ovary of a GonaCon-treated Female I. Collapsed antral follicle with cytoplasmic degeneration and granulosa layer disruption. (a) Ovary of a treated female; (b) Parenchyma, depicting a very low number of partial mature antral follicle and corpus luteum; (c) Secondary follicle with disorganized zona granulosa cells. Source: © 2019 D. Rosenfield.

**Figure 5:**
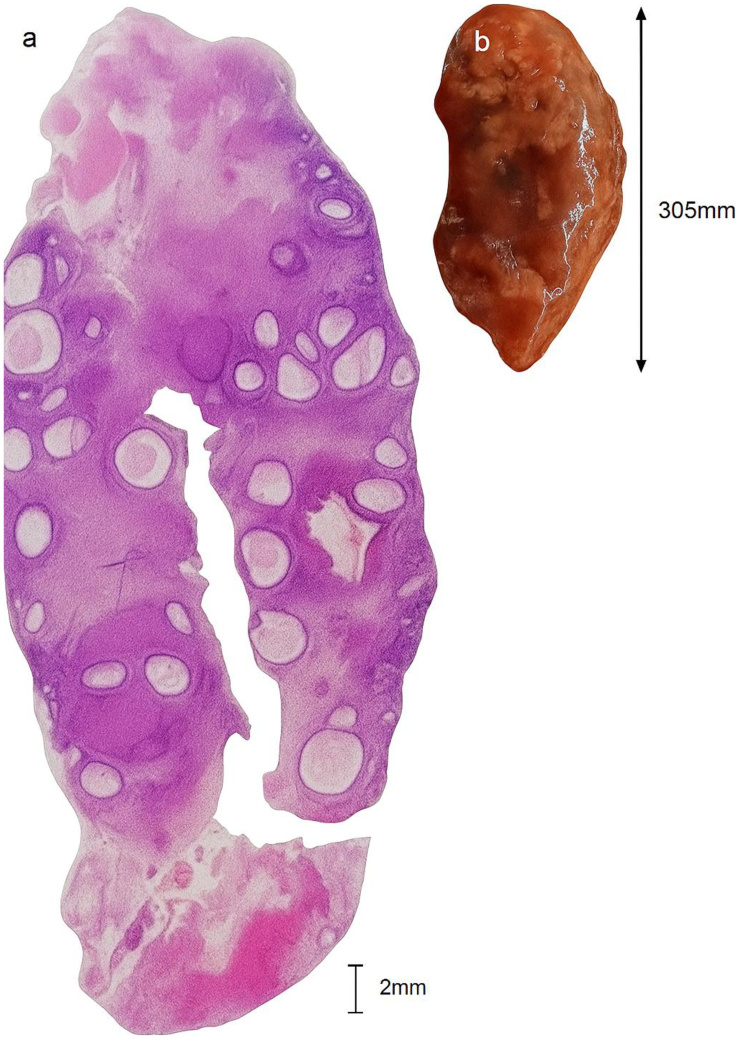
Ovary of a GonaCon-treated Female II. Longitudinal cut: the ovarian parenchyma of a GonaCon treated *Hydrochoerus hydrochaeris* female, hematoxylin-eosin staining, including (a) several antral follicles; (b) ovary with smooth surface and no protrusions. © 2019 D. Rosenfield.

**Figure 6:**
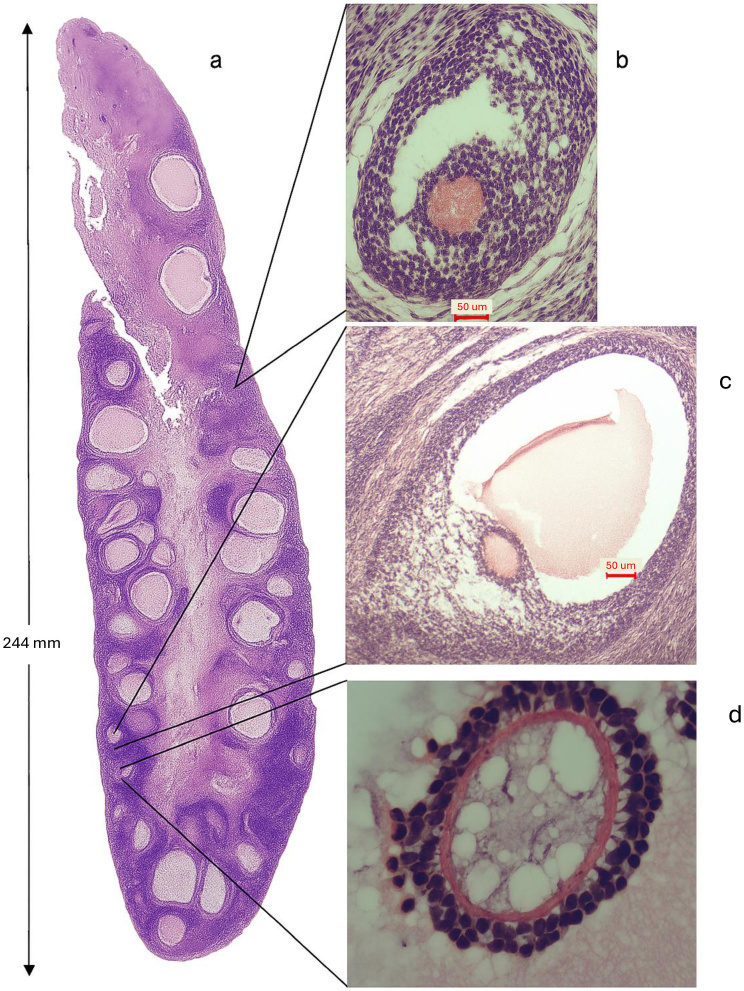
Ovary of a GonaCon-treated Female III. (a) Gross morphology of the ovary in longitudinal section showing a smooth surface and visible antral follicles; (b) pre-antral follicle (x100), with disorganized zona granulosa with formed cumulus oophorous; (c) antral follicle with present oocyte with visible inclusions within the cytoplasm, disintegrating granulosa membrane; d) oocyte with cytoplasmic dysmorphism/abnormalities. Visible corona radiata, intact zona pellucida (x400). © 2019 D. Rosenfield.

Histopathology confirmed suppressed folliculogenesis in treated ovaries: fewer antral follicles (mean 2.3 ± 0.5 SD vs. 12.7 ± 1.2 in controls; p<0.001), disorganized granulosa layers, and oocyte dysmorphism (Figures 3-6).

## Discussion

This study demonstrates that a single-dose GonaCon vaccine induces sustained fertility suppression in free-living female capybaras for ~ 33 months, with minimal welfare impacts and preserved social behaviors. Ovarian reductions and HPG axis disruption confirm the mechanism, aligning with GnRH vaccine effects in other taxa (Miller et al., 2008). Similar immunocontraceptive management outcomes have also been documented in managed ungulate populations, supporting the broader applicability of GnRH-based fertility control strategies (Bechert et al., 2022). This sustained effect appears more robust than in some rodents, such as prairie dogs, where efficacy declined after Year 1 but persisted 4-5 years in others (Shiels et al., 2024), highlighting capybara-specific immune longevity.

### Comparison with surgical contraceptive strategies

Depending on the environmental settings, financial and logistical challenges, GonaCon offers a suitable alternative to surgical methods but trades permanence for potential reversibility. These findings align with multi-year fertility suppression observed in other rodent species treated with GonaCon (Supplementary Table D).

Recent capybara studies report 100% efficacy for uterine horn ligation (Passos-Nunes et al., 2022, 2024) and OSS minimizing endocrine disruption (Jorge et al., 2024; Yanai et al., 2022). However, surgery demands high resources (anesthesia, sterility, and technical competence), less suitable for large free-ranging groups (Bechert et al., 2022). GonaCon's single-shot delivery via darting enables scalability, though boosters may be needed if efficacy wanes (Baker et al., 2023). PZP vaccines (e.g., SpayVac) provide hormone-sparing alternatives but require monitoring for boosters (Velling et al., 2025). A balanced approach, GonaCon for initial density reduction, surgery for key individuals, may optimize outcomes. Economic trade-offs favor GonaCon initially (low material cost) but surgery for long-term (no recapture); making site-specific modeling essential (ICWFC, 2022).

### Integration with one conservation framework

This work advances the One Conservation model by linking welfare (non-invasive contraception), ecosystem health (reduced tick reservoirs), and human well-being (BSF mitigation) without social disruption (Pizzutto et al., 2021). Unlike lethal culling, GonaCon preserves gregarious structures, supporting biodiversity in urban interfaces. In species where fertility returns post-GonaCon, no adverse effects on subsequent pregnancies have been observed (NWRC, 2013; Fischer et al., 2018). Efficacy varies across taxa: consistent in cervids/equids/boars but less predictable in some species (Levy et al., 2011), likewise in felids/badgers (Fischer et al., 2018; Cowan et al., 2019). Similar fertility-control approaches have also been applied in megafauna management, where reproductive suppression is used to mitigate population pressure and aggression in captive and semi-free-ranging elephants (Somgird et al., 2017). Rodent parallels (Shiels et al., 2024; Jacob et al., 2008) underscore timing's role.

### Study limitations and future directions

A key limitation in wildlife field studies, such as this one, is the small sample size (n=6 treated, n=3 control), dictated by ethical, financial, regulatory, and logistical constraints inherent to research on protected, free-ranging wildlife populations in urban environments (Rosenfield et al., 2020). While this limits the statistical power to detect small effects, the biological significance is confirmed by the consistently sustained, 100% reproductive suppression and the very large effect sizes observed in the endocrinological data (Cohen’s *d* > 1.5). These large effect sizes provide high confidence in the treatment’s robust efficacy. Looking forward, the apparent longevity of the suppression necessitates continued monitoring. Future studies must focus on tracking anti-GnRH antibody titers beyond 33 months to definitively predict the point of immunological failure or reversibility (Baker et al., 2023). Claims of humaneness are limited by the absence of non-invasive stress monitoring (e.g., FGM), which is also a rather inadequate method for capybaras due to the difficulties of individual classification. This information is important for establishing evidence-based, cost-effective re-vaccination intervals and advancing our understanding of GnRH vaccine kinetics in large wild rodents.

## Conclusion

A single intramuscular dose of the GonaCon vaccine provides sustained, safe, and humane fertility control for ~ 33 months in free-living female capybaras, with no negative impact on social behavior. This single-application method represents a scalable, welfare-oriented alternative to surgical sterilization and fulfills a critical need for reducing capybara densities to mitigate the public health threat of Brazilian Spotted Fever (BSF).

However, while GonaCon is a powerful tool, this study underscores that no single, perfect method of wildlife population control exists. Effective management requires an adaptive, integrated framework determined by the specific context: the species' biology, the desired intensity of population reduction, the geographical and regulatory location, available logistics, and public tolerance. A quantitative overview comparing these and other emerging methods across efficacy, logistics, reversibility, and behavioral impacts is provided in Supplementary Table B & C.

For complex human-wildlife issues like the capybara-BSF conflict, achieving optimal, long-term outcomes will likely require a combination of strategies, integrating advanced reproductive control (like GonaCon) with habitat modification, selective translocation, and other targeted interventions to ensure management is both effective and ethically sound.

## Data Availability

Research data is available in the body of the article. Additional methodological details, comparative analyses, and supporting citations are provided in the Supplementary Materials.

## Supplementary Material

Supplementary material accompanies this paper.

Supplementary Table A Sample Size Justification

Supplementary Table B Comparison of Common Non-Lethal Female Capybara Fertility Control Methods

Supplementary Table C Quantitative Scoring of Fertility Control Methods

Supplementary Table D Overview of GonaCon Use in Rodents

This material is available as part of the online article from: https://doi.org/10.1590/1984-3143-AR2025-0142
