# Supplementary material for: Sustained multi-year contraceptive efficacy of single-dose GonaCon in synanthropic female capybaras
Source: Anim Reprod. 2026 Apr 20;23(2):e20250142. doi: 10.1590/1984-3143-AR2025-0142 (PMC13142086; doi:10.1590/1984-3143-AR2025-0142)
Supplement: Supplementary Table A [file 1984-3143-ar-23-2-e20250142-suppl.pdf]

Supplementary Material

Supplementary Table A. Sample Size Justification

Factor	Constraint Imposed	Justification for Methodology
**Logistics & Ethics (Animal Welfare)**	**Small Sample Size (n=):** The total number of available adult, multiparous females suitable for a high-stress handling procedure (chemical immobilization and vaccination) was naturally limited in the managed environment (zoo/sanctuary).	Limiting the total number of subjects is an ethical imperative to minimize stress and risk to a captive population.
**Regulatory & Financial (Vaccine Access)**	**Limited Vaccine Availability:** The GonaCon vaccine was imported under a specific MAPA research license (LI: 16/3557599-5) Sample for research, restricting the total quantity available for the study.	The high product and freight costs as well as the strict import control of this experimental product necessitated an efficient group size that prioritized the treatment group to maximize data on efficacy.
**Statistical Efficiency (Group Ratio)**	**6:3 Group Split (Treated/Sham):** A larger proportion of the limited animals were assigned to the treatment group (6 females) than to the control group (3 females).	The primary hypothesis, that GonaCon would cause reproductive suppression, required a larger sample size in the treatment group to increase the statistical power for detecting a significant effect. The Sham group serves mainly as a contemporaneous control for handling stress and natural hormonal cycling.
**Biological Relevance (Pre-selection)**	The females were selected specifically based on being multiparous (previously reproductive), ensuring the study subjects represented the target population for field immunocontraception (reproductively active adults).	This initial selection, followed by randomization, ensured that the treatment comparison was biologically relevant, as the goal is to control population growth in established breeders.

Supplementary Table B - Comparison of Common Non-Lethal Female Capybara Fertility Control Methods

**Method**	**Surgical**	**Immunocontraception I**	**Immunocontraception II**
**Primary Mechanism**	**Ovarian Sparing Spay (OSS)**<br>Uterine Horn Ligation<br>mechanically block passage of gametes/embryos<br><br>**Ovariosalpingo-Histerectomy (OSH)**<br>Total removal of reproductive organs<br><br>**Fetal Removal**<br>Fetuses' removal w/ sterilization	**GonaCon (anti-GnRH)**<br>GnRH<br>GnRH inhibition<br>Hypothalamic-Pituitary-Gonadal (HPG) suppression	**SpayVac (PZP)**<br>Porcine Zona Prelucida<br>Oocyte-receptor – inhibiting Sperm-binding
**Hormonal Impact**	**OSS:**<br>Hormone cycle and ovaries preserved<br><br>**OSH/Fetal removal:**<br>Complete suppression	None<br>Hormonal cycles and ovaries preserved	None<br>Hormonal cycles and ovaries preserved
**Efficacy/Duration**	100%, permanent	~ 33 mo., variable	Multi-year, boosters
**Capybara tested**	Yes	Yes	No
**Invasiveness**	High (anesthesia/surgery)	Low (IM/dart)	Low (IM/dart)
**Reversibility**	**OSS:** Low/difficult<br>**OSH:** None	Moderate (antibody decay)	High (antibody decay)
**Behavioral Impact**	Minimal (hormone-sparing)	Minimal (suppression)	Minimal (sparing)
**Welfare Risk**	Moderate (infection)	Low (swelling, injection site granuloma)	Low (swelling, injection site granuloma)
**Cost/Logistics**	High initial, equipment, location, no recapture needed; Expert Competence	Low initial, potential recapture; Specialist	Low initial, potential recapture; Specialist
**Regulatory Status Brazil**	Established surgical technique	Not registered<br>Liberated for experimental use (MAPA)*	Not registered
**Key Literature**	Passos-Nunes, 2024<br>Jorge, 2024<br>Penido 2024<br>Turner, 2022<br>Yanai, 2022	Rosenfield, 2019b<br>Baker, 2023<br>Shiels, 2024	Cowan, 2019<br>Velling, 2025

* Ministry of Agriculture

Supplementary Table C - Quantitative Scoring of Fertility Control Methods

Criteria (Wish-List)	Surgical Sterilization (OSH)	Surgical Sterilization (OSS)	GnRH Immunocontraception (GonaCon)	PZP Immunocontraception (e.g., SpayVac)	Hormonal Implants (Deslorelin)
**1. Highly Effective (100% Control)**	3	3	2	3	3
**2. Long-Term Action (Multi-Year)**	3	3	2 (Variable)	1	1 (Requires Re-Dose)
**3. Single-Shot Administration**	3	3	3 (Goal of GonaCon)	1 (Often Requires Booster)	0 (Requires Re-Implant)
**4. Remote Delivery**	0	0	2	2	1
**5. Applicable to Both Sexes**	3	3	3	0 (Female Only)	2
**6. Reversible**	0	1 (High Difficulty)	2 (Variable)	2	3
**7. Minimal Adverse Effects / Pathology**	1	2	2	2	2
**8. Minimal Behavioral Impacts**	0	3 (Hormone Sparing)	2 (Avoids Total Hormone Loss)	3 (Hormone Sparing)	1 (Suppresses Endocrine Function)
**9. Logistically Feasible in Field**	1	2	3	3	2
**10. Economically Viable (Cost-Efficient)**	1	1	2	2	1
**Total Score (Max: 30)**	**15**	**23**	**23**	**19**	**15**

Footnote: Scoring 0–3 performed by the authors based on literature consensus; intended as a decision-aid tool only.

Supplementary Table D - Overview of GonaCon Use in Rodents

Rodent studies focus on free-ranging or semi-captive populations, emphasizing scalability via trapping and darting. Below, summarized key findings from peer-reviewed and EPA-registered applications, drawing from field and lab trials up to 2024.

Key Studies and Efficacy by Rodent Species

Rodent Species	Study Type & Key Details	Efficacy & Duration	Safety & Notes	Reference
**Black-tailed Prairie Dog** (*Cynomys ludovicianus*)	Field trial (2018–2020, Colorado; n=multiple colonies; 0.4 mL dose, females ≥660g targeted; 18.5% colony coverage). Replicated sites (3 treated, 3 control).	100% fertility suppression in Year 1 (juvenile:adult ratio 0.23 vs. 1.10 in controls; 3x lower juvenile density; $p=0.002$). Declined to 0% in Year 2 without full-colony treatment. EPA-registered (2022) for females; amendment pending for males.	No systemic effects; low stress via trapping. Annual boosters recommended for partial treatments.	Shiels et al. (2024); Gese et al. (2022)
**White-tailed, Gunnison's, & Black-tailed Prairie Dogs** (*Cynomys spp.*)	Captive/lab trial (2010; doses 0.1–0.4 mL, 100–400 µg GnRH conjugate; adults/juveniles). Immune response monitored 15 months.	High antibody titers at 200–400 µg doses; ≥1-year contraception predicted. No fertility return in monitoring period.	No weight loss, blood chemistry changes, or overt health issues.	Yoder & Miller (2010)
**California Ground Squirrel** (*Otospermophilus beecheyi*)	Field/captive trials (early 2000s; single injection).	≥2 years infertility; suppressed hormone production and pregnancy. Effective for urban damage control.	Minimal side effects; no toxicity noted.	Griffin et al. (2004); Nash et al. (2004)
**Fox Squirrel** (*Sciurus niger*)	Field ecology trial (2014; free-ranging).	Multi-year suppression; reduced recruitment but variable by individual. Integrated with habitat monitoring.	Safe; no behavioral disruption beyond reduced reproduction.	Krause et al. (2014) (from initial search)

Rodent Species	Study Type & Key Details	Efficacy & Duration	Safety & Notes	Reference
**Eastern Gray Squirrel** (*Sciurus carolinensis*) & **House Mouse** (*Mus musculus*)	Lab/field trials (2024; captive/wild; GnRH conjugate focus).	≥2–4 years fertility control; high efficacy in preventing litters under lab conditions. Potential for invasive species management.	Low toxicity; suitable for integrated pest management.	Jacob et al. (2024) (initial); Shiels et al. (2024)
**Norway Rat** (*Rattus norvegicus*)	Captive trial (early 2000s; single injection).	≥2 years contraception; effective in preventing breeding colonies.	No adverse health impacts; useful for commensal rodent control.	Miller et al. (various, NWRC reports)
**Capybara** (*Hydrochoerus hydrochaeris*)<br><br>Males	Field trial (2017–2019, São Paulo, Brazil; n=8 treated alpha males, n=4 controls; single 0.5 mL intramuscular dose; free-ranging synanthropic groups monitored via recaptures and observations).	100% infertility over 18 months (severe testicular atrophy, oligozoospermia, static spermatogenesis; p	No disruption to secondary sexual characteristics, courtship, or agonistic alpha-male behavior; group social integrity preserved. Transient injection-site swelling in 50% of treated males.	Rosenfield et al. (2019)

General Insights Across Rodent Applications

- **Mechanism & Delivery**: GonaCon (typically 0.4–0.5 mL intramuscular) elicits anti-GnRH antibodies, reducing progesterone/estradiol/testosterone by 30–80% within weeks. In rodents, effects onset in 1–2 months, peaking at 3–6 months. Doses scale with body size (e.g., 100–400 μg GnRH conjugate).
- **Duration Variability**: 1–2 years in small rodents (mice/rats) or partial treatments; up to 4+ years in larger ones (prairie dogs/squirrels). Factors include dose, sex (stronger in females), and coverage (full colony > partial).
- **Safety Profile**: Transient swelling (10–14 days) in ~67% of cases; no long-term toxicity, weight changes, or stress (cortisol stable). No zoonotic risks; EPA deems it safe for non-targets.
- **Management Applications**: Ideal for urban/suburban rodents (e.g., prairie dogs in Colorado parks, squirrels in agriculture). Reduces juveniles by 50–300% initially but

needs boosters for population-level impact. Complements trapping; EPA registration expanding (e.g., prairie dog label for males in 2025).

- **Limitations & Future Directions**: Small-scale trials limit generalizability; reversibility varies (not always permanent). Ongoing: Registration for more rodents (e.g., rats, squirrels); integration with rabies vaccines for feral populations. As of 2025, EPA amendments allow broader use in ground squirrels.
